# Appearance and performance factors associated with muscle building supplement use and favourable attitudes towards anabolic steroids in adolescent boys

**DOI:** 10.3389/fpsyg.2023.1241024

**Published:** 2023-09-08

**Authors:** Olivia Piplios, Zali Yager, Siân A. McLean, Scott Griffiths, Jo R. Doley

**Affiliations:** ^1^College of Health and Biomedicine, Victoria University, Melbourne, VIC, Australia; ^2^The Embrace Collective, Adelaide, SA, Australia; ^3^Institute for Health and Sport, Victoria University, Melbourne, VIC, Australia; ^4^Department of Psychology, Counselling, and Therapy, La Trobe University, Melbourne, VIC, Australia; ^5^Melbourne School of Psychological Sciences, University of Melbourne, Parkville, VIC, Australia

**Keywords:** androgenic anabolic steroids, muscle building supplements, body image, adolescent boys, sports

## Abstract

**Introduction:**

The demand for appearance and performance enhancing substances, including muscle building supplements and anabolic androgenic steroids, is increasing in Australia. However, little is known about the associations between appearance and performance-based factors and appearance and performance enhancing substances (APES), particularly among adolescent boys. This study sought to examine (a) the prevalence of muscle building supplement use in a sample of adolescent boys and (b) how both performance and appearance factors relate to muscle building supplement use and favourable attitudes towards anabolic androgenic steroids in this sample.

**Method:**

*N* = 488 adolescent boys aged 13–16 (*Mage* = 14.59) from nine Australian schools completed measures of supplement use, favourable attitudes towards using steroids, muscle dissatisfaction, body fat dissatisfaction, mesomorphic ideal internalisation, weight training, and sports participation. Hierarchical logistic regressions were used to examine cross-sectional correlates of muscle building supplement use and favourable attitudes towards using anabolic androgenic steroids.

**Results:**

In the past three months, 12.7% of the sample had used muscle building supplements. Both appearance and performance-related factors – mesomorphic ideal internalisation and weight training – were related to muscle building supplement use. Only one appearance-related factor – body dissatisfaction – was related to favourable attitudes towards anabolic androgenic steroids.

**Discussion:**

The findings from this study are important as they may help to guide intervention strategies regarding appearance and performance enhancing substance use by Australian adolescent boys, with the ultimate goal of ensuring this population’s safety.

## 1. Introduction

Appearance and performance enhancing substances (APES) including muscle building supplements (e.g., whey protein powder, creatine) and androgenic anabolic steroids (AAS) are becoming increasingly popular in Australia (Seear et al., 2015; Bowen et al., 2018). For instance, the lifetime usage of AAS doubled from 0.3% in 2004 to 0.6% in 2018 (Australian Institute of Health and Welfare, 2019). Muscle building supplements and AAS are used to increase muscle mass, reduce body fat, and improve sporting performance (Pope et al., 2014; Bowen et al., 2018). Although AAS use has consistently been associated with harmful mental and physical health outcomes (Bates et al., 2019), muscle building supplements have only recently gained attention for negative health outcomes (Butts et al., 2017; Bowen et al., 2018). One emerging concern in this field is APES use among adolescent boys (Miller et al., 2005; Dunn and White, 2011). Research has traditionally focused on adult men who use APES, but recent literature suggests adolescent boys are particularly vulnerable to APES use. For instance, Yager and McLean (2020) found 50.2% of Australian adolescent boys reported current use of APES, 10.1% had favourable attitudes towards AAS (10.1%), and 4.2% used AAS.

Adolescent boys are thought to be vulnerable to using APES due to ease of accessibility (Yager and McLean, 2020) and lack of education around usage (Miller et al., 2005; Dunn and White, 2011), but specific factors that relate APES use and favourable attitudes towards AAS are not yet clear within this population. Considering the negative health outcomes associated with the use of some muscle building supplements (Frati et al., 2015) and aggressive and/or violent tendencies associated with AAS (Kanayama et al., 2008), identifying factors that are associated with muscle building supplement use and favourable attitudes towards AAS in Australian adolescent boys is important.

It is important to note that not all muscle building supplements are harmful. Popular supplements such as protein powder and creatine are safe and effective (Australian Institute of Sport, 2022), whilst others such as testosterone boosters and pro-hormones can be dangerous (Australian Institute of Sport, 2022; Piattoly, 2022), particularly if used incorrectly. Muscle building supplements are used for several purposes; for instance, mass gainers are used for weight gain (Singh Rana and Agarwal, 2019), pro-hormones are intended to increase muscle growth and strength (Granados et al., 2014), and testosterone boosters are used to increase power and strength (Almaiman, 2018). In the current market, supplements are legally unregulated, easily accessible to the general population (Whitehouse and Lawlis, 2017), and recommended to minors by health food store employees (Herriman et al., 2017) despite manufacturers’ cautions. Of particular concern for use by young people is the fact that some muscle building supplements are associated with negative health outcomes. Mass gainers can increase blood pressure due to their high fat content (Solaković et al., 2016), pro-hormones can negatively impact cardiovascular and liver health (Granados et al., 2014) and the use of testosterone boosters is linked to kidney and liver abnormalities in some users (Almaiman, 2018). Furthermore, existing literature suggests supplement use (including supplements identified as not physically harmful) may act as a “gateway” for greater or more frequent consumption of muscle building supplements and use of products such as AAS that have higher likelihood of harm in the future (Backhouse et al., 2013; Yager and O’Dea, 2014; Lucidi et al., 2017). Given the emerging popularity of muscle building supplements in Australia and limited research regarding long-term adverse health outcomes, understanding factors that are associated with muscle building supplement use is a topic that is worthy of attention (Bowen et al., 2018).

In addition to the use of muscle building supplements, there is concern that adolescent boys are at risk of developing favourable attitudes towards using AAS (Goldberg et al., 2000). AAS are synthetic versions of testosterone and are used in clinical settings to increase muscle mass and reduce body fat (Kouri et al., 1995; Hartgens, 2004; Pope et al., 2014). However, when used outside of controlled environments, supraphysiologic dosing (a dose of a synthetic chemical that is larger than what naturally occurs in the body) can lead to negative physiological and psychological health consequences in short- and long-term users (Bates et al., 2019). Negative health outcomes associated with use can include increased aggression, depression and sexual dysfunction (Miller et al., 2005; Strother et al., 2012). Extreme side effects can include cardiovascular effects (e.g., heart attacks) which can be fatal (Frati et al., 2015). In Australia, supply and possession of AAS without a prescription is illegal, however it is relatively easy to illegally obtain AAS via the internet (Australian Government Department of Health, 2019). Further, AAS users often turn to online forums for guidance on usage techniques and dosing advice, which is often inaccurate and dangerous (Smit et al., 2019). For example, doses exceeding five times the recommended medical dosage of testosterone are frequently discussed in bodybuilding forums (Fink et al., 2019). Alarmingly, a small body of literature suggests AAS use amongst males can begin as early as adolescence (Goldberg et al., 2000), with one American study finding 7.42% of men who use AAS commenced usage prior to eighteen years of age (Bonnecaze et al., 2020). A global meta-analysis of AAS use across the lifespan found prevalence of 2.5% for adolescents aged 19 years and younger (Sagoe et al., 2014). More specifically, Australian studies have estimated prevalence of AAS use amongst adolescent boys to be 2.4% (Dunn and White, 2011), and more recently, 4.2% (Yager and McLean, 2020). These findings suggest prevalence of actual AAS usage amongst this age group is not likely to be high, and as such, the present study will instead investigate factors that are associated with favourable attitudes towards AAS. This is an important focus for research as previous research has found favourable attitudes towards AAS to predict actual AAS use later in life (Ntoumanis et al., 2014).

Across the literature on this topic, two motivational areas have consistently been associated with APES use that suggest merit for further investigation: appearance and performance-based motives (Wright et al., 2000; Yager and O’Dea, 2014). Despite being established as risk factors for APES use in adult men, factors associated with appearance or performance-based motivations have seldom been investigated in a general sample of adolescent boys, particularly within an Australian context. The current research will focus on the appearance and performance-based factors that may provide motivation to use muscle building supplements and AAS, specifically in the categories of body image (appearance based motivation) and sport participation (performance motivation).

Appearance based motivations for APES use may stem from psychological characteristics such as body dissatisfaction (Smolak et al., 2005). Body dissatisfaction is the subjective dissatisfaction with the size and/or shape of one’s body and is associated with depression and muscle dysmorphia among men (Griffiths et al., 2016). While females are typically concerned with weight loss to achieve thinness, males frequently express the dual concern of increasing muscularity and having little body fat (Strother et al., 2012; Quittkat et al., 2019). Therefore, body dissatisfaction in males is likely to be specifically concerned with body fat dissatisfaction and muscularity dissatisfaction (Jankowski et al., 2017). Body dissatisfaction can be exacerbated by multiple factors, specifically pressures from the media, family, and peers to be muscular and lean (Strother et al., 2012; Gültzow et al., 2020). Boys who feel dissatisfied with their perceived muscularity or fat levels may use exercise or diet in order to change their appearance (Smolak et al., 2005). However, adolescent boys may also turn to APES use to deal with body dissatisfaction as muscle building supplements and AAS are widely understood by the general population to increase muscularity and reduce body fat (Leone and Fetro, 2007). Body dissatisfaction is a motivating factor for APES use amongst adult men (Kanayama et al., 2020), muscle building supplement use in adolescents (Yager and McLean, 2020), and AAS use in adolescent samples (Jampel et al., 2016). Considering the dual concerns of leanness and muscularity that typify boys’ body image, the associations of both muscularity dissatisfaction and body fat dissatisfaction with APES use and attitudes will be examined.

The drive for a lean, muscular physique amongst males is born out of Western society’s mesomorphic ideal for men (McCabe et al., 2010). Internalisation of the mesomorphic ideal is where one “buys in” to the notion that a lean and muscular appearance is the ideal physique for them (Gerrard et al., 2021). Internalisation of the mesomorphic ideal has been found to influence muscularity enhancing behaviours including exercising, and APES use in adult males (Tylka, 2011). The mesomorphic body shape consists of a v-shaped torso with well-developed chest, arm and abdominal muscles (Stratton et al., 2015). Like the thin-ideal for females, the mesomorphic ideal is particularly difficult for most males (particularly adolescent males) to attain (Florescu, 2016). As such, failure to attain this ideal whilst attributing value to this ideal may result in disordered eating (Strother et al., 2012) or APES use (Jankowski et al., 2017) amongst males. Hence, exploring whether mesomorphic ideal internalisation is associated with supplement use and favourable attitudes towards AAS in Australian adolescent boys is of interest for this study.

There are dual motivations within the sporting context that may contribute towards APES use, as both amateur and professional athletes may use APES to improve (1) performance and (2) appearance (Goldberg et al., 2000). A desire to enhance performance is a factor recognised to be associated with APES use and is more common amongst amateur and recreational athletes than professionals – likely due to strict criteria around performance enhancing substances for professional athletes (Yager and McLean, 2020). However, amateur and non-amateur athletes alike may be motivated to use APES to gain a competitive “edge” on their peers (Gough, 1989). For example, both muscle building supplements and AAS have been found to enhance an athlete’s strength and improve their performance times (Sjöqvist et al., 2008). Adolescents who participate in a greater number of sports than their peers are at greater risk of engaging with APES (Goldberg et al., 2000; Dodge and Jaccard, 2006). Through the lens of performance motivations, Goldberg et al. (1996) suggest adolescents may be tempted to use APES as they strive to “win at any cost.” Further, adolescents who play sports may be at greater risk for APES use as they may be exposed to peers (other teammates) or role models (coaches) who reduce their perceived risk of negative health outcomes from APES use (Dodge and Jaccard, 2006). As such, investigating the number of sports adolescent boys’ participate in relation to their muscle building supplement use and attitudes towards AAS are of interest to this study.

Through the lens of appearance-based motivations within the sporting landscape, the relationship between sports participation and APES use may be explained by gender role conformity, a particularly salient phenomenon for adolescents (Smolak and Stein, 2010). Adolescents are particularly vulnerable to gender role conformity as they experience changes or impending changes to their bodies during puberty, as well as an increased awareness of the stereotypical roles men and women play in society (Skinner et al., 2018). Adolescent boys may aim to assert or attain masculine status through their athletic performance (e.g., muscular strength) and bodies (e.g., muscular physique; Birbeck and Drummand, 2006). Therefore, adolescent boys may be incentivised to use APES to better their athletic performance in an effort to express masculinity (Roberts et al., 2017). As such, adolescent boys who participate in more sports than their peers may be at greater risk for APES use.

In addition to sports participation, the type of sport played may influence APES use. Sports involving weight-based training (such as weightlifting and bodybuilding) have been associated with greater desire for performance enhancement than non-weight-based training sports (such as skiing, tennis, and ice hockey; Mattila et al., 2009). Additionally, weight-based sports may give athletes (both professional and amateur) dual motivations for using APES, with importance placed on both performance and appearance more than other sports (Macho et al., 2021). Bodybuilders and weight-lifting men in particular experience greater body dissatisfaction and increased drive for muscularity than men who do not engage in these sports (Olivardia et al., 2000; Schneider et al., 2019). Weight training often occurs in the gym, an environment that can influence use of and favourable attitudes towards APES (Bates, 2019). Gym environments have been found to normalise use of and have favourable attitudes towards APES (Dennington et al., 2008). Weightlifters may receive praise or approval from their peers if they engage in or approve of APES use, which may therefore act as positive reinforcement for usage (Bates et al., 2019). During adolescence, people are particularly concerned with gaining peer approval and social acceptance from others (Gruenenfelder-Steiger et al., 2016). Therefore, adolescents entering the gym environment or joining the weight-lifting community may be at heightened risk for engagement with APES, as they may gain social reward in using or having positive attitudes towards supplements and/or AAS. As such, further research investigating the relationship between weight training and supplement use and favourable attitudes towards AAS is warranted.

Existing literature suggests there is a demand for muscle building supplements and AAS within Western countries (Dunn and White, 2011; Sagoe et al., 2014; Yager and McLean, 2020; Cunningham and Griffiths, 2021). However, our understanding of APES behaviours in Australian adolescent boys, particularly factors that are associated with supplement use and favourable attitudes towards AAS, is still in its infancy. Expanding knowledge in this area is relevant as previous intervention strategies have yielded inconsistent results (Lucidi et al., 2017; Yager et al., 2019, 2023). Therefore, the present study’s exploration of factors related to the use of and favourable attitudes towards APES such as body dissatisfaction, mesomorphic ideal internalisation and sports engagement may help to further the literature in this area. By enhancing understanding of attitudes and behaviours towards APES use in adolescent boys, the present study has the potential to provide an important framework that future intervention strategies can draw from to more effectively target at-risk males.

As such, this study has two aims. The first is to estimate the rate of muscle building supplement use in a sample of Australian boys aged 13–16 years. The second is to understand how body image and sport participation are associated with adolescent boys’ (a) use of supplements and (b) favourable attitudes towards AAS. The hypotheses that will be tested are as follows:

1. Higher levels of muscularity dissatisfaction, body fat dissatisfaction, and thin-ideal internalisation will be positively and significantly related to (a) muscle building supplement use and (b) favourable attitudes towards AAS.
2. The number of sports boys participate in, as well as participation in sports involving weight-based training, will be significantly and positively related to (a) muscle building supplement use and (b) favourable attitudes towards AAS.

## 2. Method

### 2.1. Research design

The present study is part of a larger Randomised Controlled Trial of a school-based body image program called “Goodform” (Yager et al., 2023). The study protocol for the intervention is described in detail in Doley et al. (2020). The current study focuses specifically on factors related to muscle building supplement use and favourable attitudes towards AAS prior to intervention – i.e., at baseline, using a cross sectional design.

### 2.2. Participants

Participants were *N* = 512 year 9 and 10 boys (aged 13–16, *Mage* = 14.59, SD*age* = 1.95) attending secondary school in Australia. Data were collected from nine schools in urban and rural regions from the states of Victoria, New South Wales, Queensland, and South Australia. Participants were males attending year 9 or 10 at an Australian secondary school that agreed to facilitate the program. At the school level of eligibility, the school was required to deliver Goodform in a single-sex setting, e.g., an all boys’ school, or during single sex health and physical education classes. There were no exclusion criteria.

### 2.3. Measures

In the Goodform trial, participants completed questionnaires relating to APES and body image. The subsection of measures used for the current research are described below. All measures were self-reported and completed online using the survey software Qualtrics. The Simple Measure of Gobbledygook (McLaughlin, 1969), found the questionnaire to be suitable for early adolescent reading levels.

#### 2.3.1. Demographic variables

Demographic variables were collected by asking participants to record their date of birth and postcode from which age (relative to survey date) and geographic location were derived. Locations based on postcodeswere coded as Major cities =1, Inner regional = 2, outer regional = 3, remote Australia = 4, very remote Australia = 5, whereby higher scores indicated greater remoteness of location in accordance with the Australian Statistical Geographical Standard (ASGS) guidelines, edition 2 (Australian Bureau of Statistics, 2016). If boys did not provide their postcode, their school postcode was used to indicate location in accordance with the ASGS.

#### 2.3.2. Body fat dissatisfaction and muscularity dissatisfaction

The Male Body Attitudes Scale (MBAS) assessed body dissatisfaction using the subscales of muscularity dissatisfaction (7 items) and body fat dissatisfaction (5 items; Tylka et al., 2005). Responses to items from both subscales were recorded on a 5-point Likert-type scale from 1 (Never) to 5 (Always). The mean for each subscale was used for analyses, with higher scores reflecting greater body dissatisfaction. For the muscularity dissatisfaction subscale, an example item is: “I think I have too little muscle on my body” (Tylka et al., 2005). For the body fat dissatisfaction subscale, an example item is: “I think my body should be leaner” (Tylka et al., 2005). Each subscale had good internal consistency, with Cronbach’s *a* = 0.86 (muscularity dissatisfaction), and Cronbach’s *a* = 0.88 (body fat dissatisfaction). These metrics were consistent with previous research in adolescent males (Sepúlveda et al., 2016).

#### 2.3.3. Muscular-ideal internalisation

The muscularity internalisation subscale from the Sociocultural Attitudes Towards Appearance Questionnaire-4-Revised (SATAQ-4-R; Schaefer et al., 2015) was used to assess mesomorphic ideal internalisation. The subscale contains four items such as, “It is important for me to look muscular,” with responses recorded on a 5-point Likert-type scale ranging from 1 (Definitely Disagree) to 5 (Definitely Agree). The mean score was used for analyses, with higher scores indicating greater internalisation of the mesomorphic ideal. Cronbach’s alpha was *a* = 0.87, which evidenced good internal consistency, and was in line with previous research with persons under eighteen (Neves et al., 2020).

#### 2.3.4. Sports participation

To evaluate sports participation, boys were asked to list the sports they currently played. Responses were provided in an open text format, with participants asked to type their response in a box. Answers for each respondent were then tallied following data collection, where a singular numerical value was recorded and used for analysis. For example, a student who listed they played “volleyball, AFL and soccer” received a score of 3. This approach has proven to be a successful measure in previous research, as evidenced by Yager and McLean (2020).

#### 2.3.5. Weight training sports

To assess participation in weight-based training, a dichotomous variable was created post data collection to indicate whether students engaged in weight training. The variable was created based on the open responses that participants provided to indicate sports participation described above. Participants who reported engaging in sports involving weight-based training (example responses included “weights” and “gym”) were identified and given a score of “1”. Participants who only listed sports that do not directly involve weight training were given a score of “0”.

#### 2.3.6. Supplement use

To assess the dependent variable of supplement use in adolescent boys, participants were asked if they had used muscle building supplements in the past three months using the wording *“Have you used supplements to build muscles or burn fat? These are things like protein powder, creatine, testosterone booster, or fat burners like green tea extract.”* Answers of “yes” (scored as 1) or “no” (scored as 0) were recorded.

#### 2.3.7. Attitudes towards using steroids

Finally, to assess the dependent variable of favourable attitudes towards using AAS, items from the Attitudes Towards Using Anabolic Steroids: Outcome Expectations For Using Steroids (OE-AAS) and Intentions to Use Steroids (I-AAS) were used (Parent and Moradi, 2011). Responses to items are reported on a 7 point Likert scale from 1 (Strongly Agree) to 7 (Strongly Disagree), with higher scores indicating less favourable attitudes towards AAS. Responses to items were combined to form a single scale. An example item from the OE-ASS is “If I used anabolic AAS, I would be more confident” (Parent and Moradi, 2011). An example item from the I-AAS is “I plan to use anabolic steroids in the future” (Parent and Moradi, 2011). Cronbach’s alpha indicated excellent internal consistency, with *a* = 0.96.

### 2.4. Procedure

The research was approved by the Victoria University Human Research Ethics Committee (HREC; HRE: 18:175), the Victorian Department of Education and Training (2018_003920), and NSW Department of Education and Training (SERAP 2020406). Participants were recruited via their schools. A member of the research team contacted school principals or physical education teachers via phone or email to invite school participation. The email or phone call described the nature of the project, including an overview of background literature. If willing to participate, principals and teachers provided consent for their school’s participation via an electronic consent form. To minimise administrative work for the school, an informed opt-out consent process was used for adolescents and their parents. Three weeks prior to data collection, parents and adolescent boys received comprehensive information about the study in the form of information sheets and explanatory videos. Parents and adolescents were encouraged to contact the research team with any queries prior to commencement. To opt their child out of the research, parents completed an electronic consent form via the survey software Qualtrics. Boys from public schools were required to sign an electronic consent form, as required by ethics protocols. Boys from independent schools indicated their consent by returning the completed questionnaire. Participants could withdraw consent at any time by exiting the survey or notifying the research team.

Surveys were completed in Health and Physical Education classes at school under teacher supervision. On average, participants took approximately 15 min to complete the survey. Surveys were completed in exam-like conditions to ensure privacy for participants. Additionally, a list of support contacts were made available to participants following completion of the questionnaire, including a clinical psychologist from the research team. Each school received a $200 gift card to thank them for taking part in the research.

### 2.5. Data analysis

All data were analysed using IBM SPSS version 29. Data checks were conducted to examine the shape of the distributions and potential outliers. Outliers due to human error (e.g., age = 100) were identified and deleted. Responses that were deemed inappropriate or silly (i.e., indicating that the participant was not taking the survey seriously) were identified by a member of the research team, and two additional team members provided their agreement on whether or not the participant’s responses should be excluded from analysis. A total of 20 cases were identified as inappropriate responders at baseline, and thus excluded from analysis. One case who completed the survey too quickly (less than 180 s) was excluded from analysis. Three cases were excluded for answering less than 5% of the survey questions. The final sample comprised *n* = 488 participants.

Prevalence of muscle building supplement use within the current sample was examined with frequencies. To explore hypotheses, a logistic regression was used, with an alpha level of 0.05, to assess factors associated with of supplement use. As favourable attitudes towards AAS was highly negatively skewed (as higher scores indicated less favourable attitudes towards using AAS – see Supplementary material 1), a gamma regression was planned. However, upon inspections of scatterplots, q-q plots, and histograms of the residuals, it was evident that assumptions of multivariate normality and homoscedasticity were not met (See Supplementary material 2). As such, favourable attitudes towards AAS was examined as a dichotomous variable using a logistic regression. To transform favourable attitudes towards AAS into a dichotomous variable, mean scores less than 5 were coded as 1, indicating some favourable attitudes towards AAS, and mean scores equal to or greater than 5 were coded as 0, indicating no favourable attitudes towards AAS. This cut-off point was chosen as 5 on the scale indicated “somewhat disagree” (indicating overall unfavourable attitudes towards AAS use).

Both logistic regressions included the independent variables of muscle dissatisfaction, body fat dissatisfaction, mesomorphic ideal internalisation, number of sports played, and weight training participation. To examine missing data, Little’s MCAR test was used, which yielded a significant result of χ^2^ (15) = 27.12, *p* = .028 (for the muscle building supplement analysis) and χ^2^ (15) = 26.57, *p* = .036 (for the favourable attitudes towards AAS analysis), denoting that data were not missing completely at random. Listwise deletion was used as the remaining data were deemed to be adequately powered; according to Peduzzi et al. (1996) approximately 10 events per predictor are needed for adequate statistical power for logistic regression. There were 5 predictors for each regression, with 54 events for supplement use, and 75 events for favourable attitudes towards AAS.

## 3. Results

### 3.1. Preliminary analyses

The descriptive statistics and bivariate correlations between variables used in all analyses are displayed in Table 1. Correlations were in the expected directions. Participants ranged in age from 13.22–16.86 years old, *M =* 14.81, SD = 0.50. The majority of participants lived in major cities (46.9%), followed by inner regional areas (40.5%), and outer regional areas (12.7%).

**Table 1:** Descriptive statistics and correlations for variables included in analyses.

	*N*	*M (SD)*	Range or %	1	2	3	4	5	6	7
1. MD	474	2.35 (0.83)	1–5	–						
2. BFD	475	2.00 (1.03)	1–5	.45***	–					
3. MII	481	3.22 (0.91)	1–5	.46***	.16***^†^	–				
4. No. sports	466	2.40 (1.38)	0–17	.11**	−.06^†^	.13**	–			
5. Weight training^a^	466	–	15.2	.15***	.13**	.26***	.28**	–		
6. Supplement use^b^	472	–	12.7	.16***	.15***	.19***	.13**	.19***	–	
7. Attitudes towards AAS^c^	477	–	17.0	.18***	.19***	.14**	.06	.03	.21***	–

^a^% indicates those who engaged in weight training, ^b^% indicates those who used supplements, ^c^% indicates those who have positive attitudes towards AAS use, ^†^indicates Spearman correlation was used, MD, muscularity dissatisfaction; BFD, body fat dissatisfaction; MII, mesomorphic ideal internalisation. * = *p* < 0.05, ** = *p* < 0.01, *** = *p* < 0.001.

### 3.2. Sample prevalence of muscle building supplement use

As displayed in Table 1, the prevalence of muscle building supplement use within the sample was 12.7%.

### 3.3. Factors associated with muscle building supplement use

Assumptions for the linearity of the logit were met as no significant interaction effects were found between independent variables and their logs. Multicollinearity was assessed by inspecting the VIF (<10) and tolerance (>0.10) for each independent variable, where the assumption was met for all independent variables. To assess outliers, leverage statistics, Cook’s distance, and dfBetas were inspected. A holistic approach was taken to assessing outliers as suggested by Field (2009), specifically examining casewise diagnostics in context. Cases with simultaneously high leverage values [defined as >2(k + 1/*n*)], high dfbetas (defined as >2/ √(n)) , and Cook’s distance >1 that could not be explained within the context of the data were removed from analyses. No cases were found to be problematic, and as such all were retained in the final sample. All other assumptions including multicollinearity, linearity of the logit and residual testing were met and analyses proceeded.

To examine factors associated with supplement use, a logistic with the factors number of sports played, muscle dissatisfaction, body fat dissatisfaction, mesomorphic ideal internalisation and weight training was conducted. The results of the regression are displayed in Table 2.

**Table 2:** Logistic regression examining correlates of muscle building supplement use.

Predictor	ẞ	SE (ẞ)	Wald’s χ^2^	*df*	*p*	eẞ (Odds Ratio)
No. sports played	0.08	0.09	0.80	1	.372	1.08
Muscle dissatisfaction	0.20	0.22	0.80	1	.370	1.22
Body fat dissatisfaction	0.23	0.15	2.28	1	.131	1.26
Mesomorphic ideal internalisation	0.43	0.22	3.88	1	.049	1.53
Weight training	0.95	0.36	6.93	1	.008	2.57

*n* = 447.

Hosmer and Lemeshow tests found that the data were a good fit for the logistic model, *p* = .360. The overall model did not indicate good fit to the data for Nagelkerke R square = .13, or Cox & Snell R square = .07 (Hemmert et al., 2018). However, the model was significantly related to the outcome of muscle building supplement use, χ^2^ (5) = 32.34, *p* < .001. Mesomorphic ideal internalisation was a significant, positive correlate of supplement use, *B* = 0.43, Wald χ^2^ = 3.88, *p* = .049. For each unit increase in mesomorphic ideal internalisation, the odds of using muscle building supplements increased by 53%, Odds Ratio = 1.62, 95% CI [1.00, 2.34]. Additionally, weight training was a significant positive correlate of supplement use, *B* = 0.95, Wald χ^2^ = 6.93, *p* = .008. For those who engaged in weight training, the odds of using muscle building supplements increased by 157%, Odds Ratio = 2.57, 95% CI [1.27, 5.20] relative to those who did not engage in weight training. Muscularity dissatisfaction, body fat dissatisfaction, and number of sports played were not found to be significantly associated with supplement use in the model.

### 3.4. Factors associated with favourable attitudes towards steroids

To examine factors associated with favourable attitudes towards AAS, a logistic regression with the independent variables of number of sports played, muscle dissatisfaction, body fat dissatisfaction, mesomorphic ideal internalisation and weight training was conducted. Assumptions of linearity of the logit, residuals and multicollinearity were tested, with all being satisfied. Outliers were identified using the same procedure as for the previous regression; none met the criteria and as such were retained.

Hosmer and Lemeshow tests found that the data were a good fit for the model, *p* = .271. The overall model did not indicate good fit to the data according to Hemmert et al. (2018); Cox & Snell R square = .05 and Nagelkerke R square = .09, but the overall model was significant, χ^2^(5) = 23.10, *p* < .001. Body fat dissatisfaction was a significant positive correlate of favourable attitudes towards AAS, *B* = 0.35, Wald’s χ^2^ = 7.31, *p* = .007. For each unit increase in body fat dissatisfaction, favourable attitudes towards using AAS increased by 42%; Odds Ratio = 1.42, 95% CI [1.10, 1.84].

Muscularity dissatisfaction, mesomorphic ideal internalisation, weight training and number of sports played were not found to be significantly associated with attitudes towards using AAS in adolescent boys (see Table 3).

**Table 3:** Logistic regression examining favourable attitudes towards steroids.

Predictor	ẞ	SE(ẞ)	Wald’s χ^2^	*df*	*p*	eẞ (Odds Ratio)
No. sports played	0.09	0.08	1.32	1	.250	1.10
Muscle dissatisfaction	0.18	0.19	0.87	1	.350	1.19
Body fat dissatisfaction	0.35	0.13	7.31	1	.007	1.42
Mesomorphic ideal internalisation	0.31	0.18	3.02	1	.082	1.36
Weight training	−0.26	0.37	0.48	1	.485	0.77

*n* = 444.

## 4. Discussion

The present study had two aims. The first was to estimate the prevalence of muscle building supplement use in a sample of Australian adolescent boys aged 13–16 years. It was found that 12.7% of boys used muscle building supplements within this sample. The second aim was to understand how body image and sports participation are associated with adolescent boys’ supplement usage and their favourable attitudes towards AAS. The first hypothesis, that higher levels of muscularity dissatisfaction, body fat dissatisfaction and mesomorphic ideal internalisation would be significantly related to supplement use and favourable attitudes towards AAS was partially supported, as only mesomorphic ideal internalisation was significantly related to supplement use, and only body fat dissatisfaction was significantly related to favourable attitudes towards AAS. It was also hypothesised that frequency of sport participation and participation in weight-based training would be significantly related to muscle building supplement use and favourable attitudes towards AAS. This second hypothesis was also partially supported, as only weight training was associated with muscle building supplement use. Overall, it appears that both appearance and performance-related factors are associated with muscle building supplement use, while only appearance-related factors are associated with favourable attitudes towards AAS. However, not all hypothesized dimensions of these motivations were associated with these outcomes within multivariate models.

### 4.1. Sample prevalence of muscle building supplement use

The number of boys using muscle building supplements was lower than expected in the present study’s sample, with 12.7% of participants reporting muscle building supplement use in the past three months. This was surprising, as previous research recently conducted by Yager and McLean (2020) found 49.8% of adolescent boys currently used muscle building supplements. The present study’s sample consisted of participants from both public and private schools from both rural and urban areas, whereas Yager and McLean (2020)’s study examined boys from a single urban private school. Due to the costs typically associated with muscle building supplement use, this may contribute to the lower prevalence of use in this sample compared to previous research (Yager and McLean, 2020). Additionally, as Yager and McLean (2020)’s study asked about having *ever* used muscle building supplements rather than within the past three months, this may explain the discrepancy; regular or continued use of muscle building supplements may be less common than single or irregular use.

### 4.2. Correlates of muscle building supplement use

Greater mesomorphic ideal internalization was associated with supplement use, which was consistent with previous research on contributors to muscle building behaviours (Tylka et al., 2015; Rodgers et al., 2020). However, it is particularly interesting to note that body fat and muscularity dissatisfaction were not significantly associated with muscle building supplement use, but the internalization of unrealistic appearance ideals – i.e., “buying in” to the importance of appearing lean and muscular – was. Essentially, feeling dissatisfied in one’s appearance is not sufficiently salient to be associated with muscle building supplement engagement, but holding up the ideal of muscularity as important is. This is concerning as images of the mesomorphic ideal are ever present in our society, featuring heavily across social media platforms, film and television, and print media (Gerrard et al., 2021). As the consumption of media is relatively high amongst adolescent boys (Statista Research Department, 2022a,b), their exposure to the mesomorphic ideal across multiple media channels may be hard to regulate. Therefore, the findings of the present study indicate the necessity to identify buffers against mesomorphic ideal exposure to prevent or reduce internalisation. Protective factors, such as self-compassion, which have been found to moderate the relationship between thin ideal internalisation and disordered eating in women (Tylka et al., 2015) may also be relevant in the present context. Hence, future research may wish to examine the effects of self-compassion as a buffer to mesomorphic ideal internalisation in adolescent boys, and further examine whether this influences use of muscle building supplements.

Additionally, weight training was related to supplement use, which is consistent with Yager and McLean (2020). Considering muscle building supplements are marketed specifically to aid in improving recovery from exercise, as well as to increase muscle size, it is unsurprising that their use was greater among those who engaged in weight-based training. Muscle building supplement use is particularly normalized within environments that include weight-based training (Dennington et al., 2008; Bates et al., 2019). Although supplements such as protein powder and creatine are relatively benign, there is concern that they may act as a “gateway” for more serious muscle building supplement and AAS use (Yager and O’Dea, 2014; Lucidi et al., 2017); as such, environments that facilitate weight training for adolescents may wish to be mindful that they do not normalize their use for young people in particular.

Our finding that the number of sports played was unrelated to supplement use is inconsistent with Yager and McLean (2020) who found that adolescent boys who participated in a greater number of sports were more likely to use muscle building supplements. While this variable was related at the univariate level, the relationship was not retained when controlling for other performance and appearance factors. A possible explanation for our failure to replicate Yager and McLean (2020)’s finding lies within the type of supplement use examined; that is, their research asked about lifetime supplement use, whereas ours examined use within the past three months. Future research may wish to investigate whether this finding can be replicated in another study examining recent, rather than lifetime, supplement use.

Surprisingly, both body fat and muscularity dissatisfaction were unrelated to supplement use in the logistic regression, which is inconsistent with previous research (Kanayama et al., 2020; Yager and McLean, 2020), although they were related at the univariate level when other variables were uncontrolled for. The low usage of muscle building supplements amongst the current study’s sample (12.7%) may explain these unexpected results. Muscularity dissatisfaction within the sample was somewhat more normative. As such, perhaps muscularity dissatisfaction is more likely to be associated with muscle building supplement use or favourable attitudes towards AAS at a clinical level or an otherwise at risk sample (e.g., young athletes) rather in a universal sample. Additionally, muscle building supplements may be better associated with muscularity related gains rather than body fat loss, which would explain the absence of a relationship between body fat dissatisfaction and supplement use, particularly in the multivariate model.

### 4.3. Correlates of favourable attitudes towards AAS

The sole factor associated with favourable attitudes towards AAS was body fat dissatisfaction. It is notable that only an appearance related factor was related to this outcome rather than a combination of appearance and performance factors. The association of appearance-based factors with favourable attitudes towards AAS is consistent with previous literature, which finds that favourable attitudes towards AAS use are linked with a desire for leanness (Jampel et al., 2016; Kanayama et al., 2020; Yager and McLean, 2020), and specifically have appearance-based motivations for use, in addition to performance-based motivations. The significance of body fat dissatisfaction in the model implies that the desire for leanness should not be ignored when assessing vulnerability to AAS use; although muscularity related motivations have previously been identified as important, it appears that assessment of multiple domains of body image are important to examine for adolescent males.

Surprisingly, muscularity dissatisfaction, mesomorphic ideal internalization, weight training, and number of sports played were unrelated to favourable attitudes towards AAS. Similar explanations to our unexpected findings for muscle building supplements are offered; specifically, sample-based factors may explain our results. Muscularity dissatisfaction, mesomorphic ideal internalization, weight training, and number of sports played may be relevant to AAS attitudes among groups at greater risk; for example, athletes. Favourable attitudes towards AAS were low (17.0%) in the current sample, which may support this explanation. Furthermore, previous research examining predictors or correlates of AAS is mostly based on older adolescent or adult samples (e.g., Dunn et al., 2009; Kanayama et al., 2020); there may be less understanding around AAS at a younger age group. Future research may wish to examine whether these factors predict AAS use in higher risk samples of early to mid-adolescents. It is also interesting to note that both muscular-ideal internalization and muscularity dissatisfaction were correlated with favourable attitudes towards AAS at the univariate level but not in the multivariate models; body fat dissatisfaction emerged as the correlate with greater shared variance and should be considered in future models of supplement and AAS intentions and use. Finally, early to mid adolescents may not associate AAS use with performance gains for their level of sport participation, which would explain why performance related factors are not associated with favourable attitudes towards AAS use among this sample.

### 4.4. Clinical and theoretical implications

The results of the present study are beneficial as they provide valuable insight into the attitudes and behaviours toward APES amongst Australian adolescent boys; specifically as they demonstrate that muscular ideal internalization and weight training are associated with supplement use, and body fat dissatisfaction is associated with favourable attitudes towards AAS in adolescent boys. To the authors’ knowledge, the current body of literature is limited in this area and further research expanding our understanding on the topic is essential for reducing harms from APES use in adolescent boys. The outcomes of the present study help to consolidate findings from previous research (e.g., Tylka, 2011; Jankowski et al., 2017; Rodgers et al., 2020; Yager and McLean, 2020), identifying the associations mesomorphic ideal internalisation, body fat dissatisfaction and weight training have with use of and positive attitudes towards APES. It is important to note that both appearance and performance factors were associated with muscle building supplement use, while only appearance related factors were associated with favourable attitudes towards AAS. This finding has the practical implication that both appearance and performance factors should form the basis of prevention and intervention for adolescent boys. A need for intervention direction is relevant given previous universal intervention attempts that did not lead to significant improvements on outcome measures (Yager et al., 2023).The current study’s findings suggest that targeting programs towards boys who experience body fat dissatisfaction, have higher levels of internalisation of the muscular “ideals”, and/or engage in weight training may yield better results.

### 4.5. Strengths and limitations

A major strength of the present study was the vast cohort of participants who comprised the sample. By collecting data from participants in several locations around Australia, including both regional and urban communities, the present study provides a more accurate and diverse insight into the attitudes and behaviours towards APES amongst Australian adolescent boys than previous research. However, as data were collected in varied geographic locations, during the Covid pandemic when researchers were not permitted in schools, data collection was not supervised by the researchers, which may have reduced the data quality. Furthermore, our study only explored prevalence of use within the current sample, as the dearth of literature on muscle-building supplement use among adolescents makes precise power calculation estimates difficult.

A limitation of the present study was the broadness by which “weight training” in participants was identified. Participants were not directly asked whether they engage in weight training, rather this information was inferred from recorded responses of sports participation. As such, there may have been participants who played sports such as Australian Rules Football, which may involve weight training as part of general training, but went undetected in the present study. In their study, Yager and McLean (2020) specifically asked participants if they engaged in weight training whereby responses of “yes” or “no” were recorded. Future research may wish to implement this strategy to investigate whether weight training predicts muscle building supplement use and favourable attitudes towards AAS. A further limitation of our study is that our research design is cross-sectional; as such, the directionality of our results is inconclusive. Longitudinal research would benefit this area, in order to identify specific risk and/or protective factors for muscle-building supplement use and favourable attitudes towards AAS.

## 5. Conclusion

A combination of both appearance (mesomorphic ideal internalization) and performance (weight training) factors are related to muscle building supplement use in adolescent boys, whereas only appearance motivations (body fat dissatisfaction) were related to favourable attitudes towards AAS in adolescent boys. However, somewhat encouragingly, muscle-building supplement use was lower than in previous research. Despite the low prevalence, our findings indicate the need for interventions that improve body image in adolescent boys, while also ensuring that sporting and gym environments avoid encouraging APES use. It is essential that further research is conducted on this topic due to the rise of APES use in Australia, and the dangers associated with consumption of muscle-building supplements. In particular, longitudinal research on predictors of muscle-building supplement use and favourable attitudes towards using AAS can help to guide and target strategic interventions for Australian adolescent boys, working toward the ultimate goal of preventing harms to this population.

## Data availability statement

Data will not be available to the public, as ethical approvals did not include data availability, and as participants are under 18, so we wish to maintain total privacy and confidentiality of the data. Any researchers conducting meta-analyses can contact final author SG for access to de-identified data on request. Requests to access the datasets should be directed to SG; scott.griffiths@unimelb.edu.au.

## Ethics statement

The Goodform trial was approved by the Victoria University Human Research Ethics Committee (approval number: HRE18-027), and the NSW (approval number: SERAP 2020406), and Victorian (approval number: 2018_003920) Department of Education Ethics Committees. The studies were conducted in accordance with the local legislation and institutional requirements. The ethics committee/institutional review board waived the requirement of written informed consent for participation from the participants or the participants’ legal guardians/next of kin for independent schools, as parents provided written informed dissent.

## Author contributions

OP, ZY, SM, SG, and JD assisted in writing this manuscript, with OP writing all initial drafts for all sections of the manuscript. JD and OP completed the data analyses. ZY developed the concept for this project. OP, ZY, SM, SG, and JD contributed to study design. All authors contributed to the article and approved the submitted version.

## Funding

This project is funded by a grant from the World Anti-Doping Agency. The funding body has no role in design of the study and collection, analysis, and interpretation of data or in writing the manuscript.

## Conflict of interest

The authors declare that the research was conducted in the absence of any commercial or financial relationships that could be construed as a potential conflict of interest.

## Publisher’s note

All claims expressed in this article are solely those of the authors and do not necessarily represent those of their affiliated organizations, or those of the publisher, the editors and the reviewers. Any product that may be evaluated in this article, or claim that may be made by its manufacturer, is not guaranteed or endorsed by the publisher.
