# Supplementary material for: Appearance and performance factors associated with muscle building supplement use and favourable attitudes towards anabolic steroids in adolescent boys
Source: Front Psychol. 2023 Sep 8;14:1241024. doi: 10.3389/fpsyg.2023.1241024 (PMC10516554; doi:10.3389/fpsyg.2023.1241024)
Supplement: Supplementary file 1 [file Data_Sheet_1.pdf]

Explore

Notes

Output Created		30-APR-2023 08:26:06
Comments		
Input	Data	/Users/joannadoley_1/Dropbox/Shared Folder (2)/Data/Data for Olivia/JoDataredo.sav
	Active Dataset	DataSet2
	Filter	<none>
	Weight	<none>
	Split File	<none>
	N of Rows in Working Data File	491
Missing Value Handling	Definition of Missing	User-defined missing values for dependent variables are treated as missing.
	Cases Used	Statistics are based on cases with no missing values for any dependent variable or factor used.
Syntax		EXAMINE VARIABLES=AttAASTotal

		/PLOT BOXPLOT HISTOGRAM NPPLOT<br>/COMPARE GROUPS<br>/STATISTICS DESCRIPTIVES<br>/CINTERVAL 95<br>/MISSING LISTWISE<br>/NOTOTAL.
Resources	Processor Time	00:00:00.48
	Elapsed Time	00:00:01.00

Case Processing Summary

	Cases					
	Valid		Missing		Total	
	N	Percent	N	Percent	N	Percent
AttAASTotal	477	97.1%	14	2.9%	491	100.0%

Descriptives

			Statistic	Std. Error
AttAASTotal	Mean		6.036105	.0639006
	95% Confidence Interval for Mean	Lower Bound	5.910543	
		Upper Bound	6.161667	
	5% Trimmed Mean		6.226920	
	Median		6.666667	
	Variance		1.948	
	Std. Deviation		1.3956106	
	Minimum		1.0000	
	Maximum		7.0000	
	Range		6.0000	

	Interquartile Range	1.3889	
	Skewness	-1.963	.112
	Kurtosis	3.823	.223

Tests of Normality

	Kolmogorov-Smirnov[a]			Shapiro-Wilk		
	Statistic	df	Sig.	Statistic	df	Sig.
AttAASTotal	.245	477	<.001	.720	477	<.001

a. Lilliefors Significance Correction

AttAASTotal

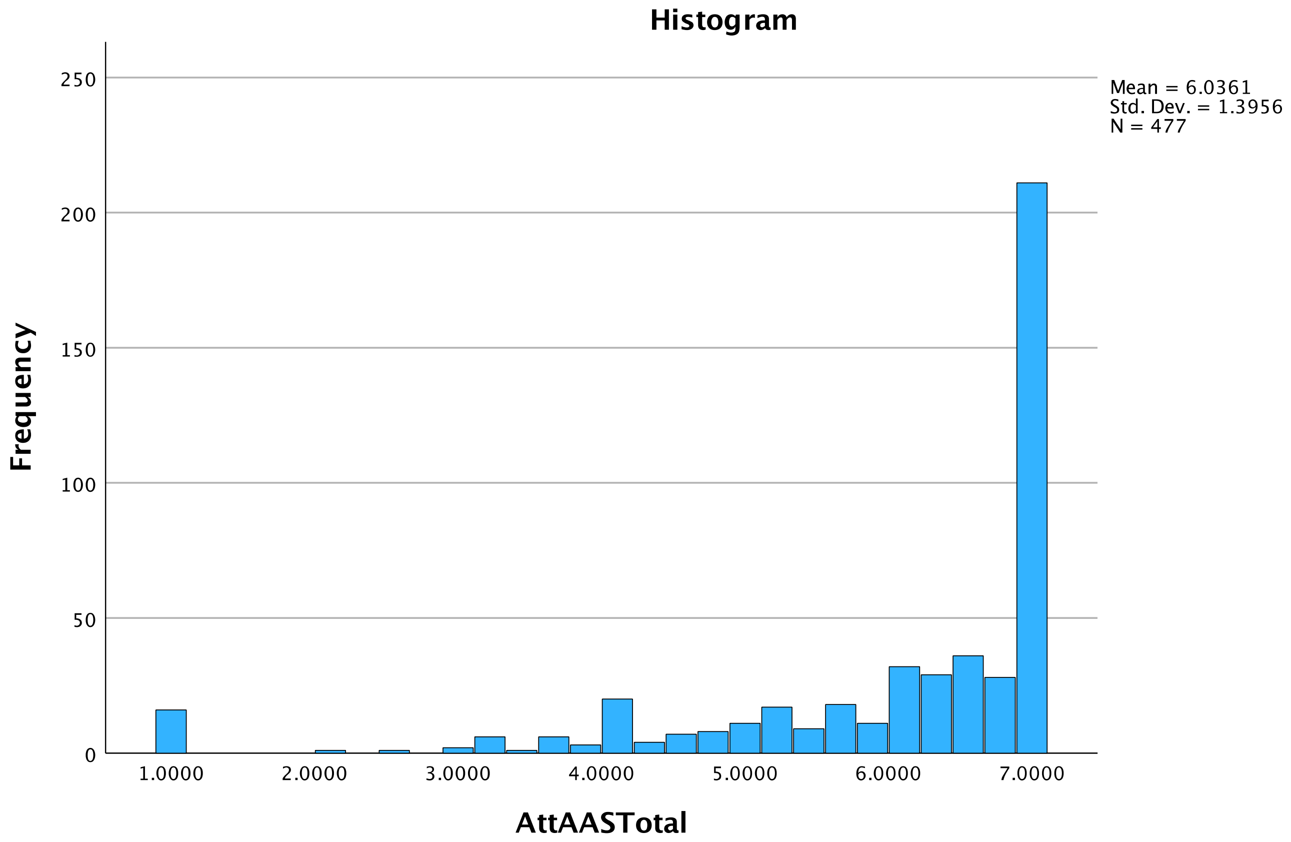
Histogram
Mean = 6.0361
Std. Dev. = 1.3956
N = 477
Frequency
250
200
150
100
50
0
1.0000
2.0000
3.0000
4.0000
5.0000
6.0000
7.0000
AttAASTotal

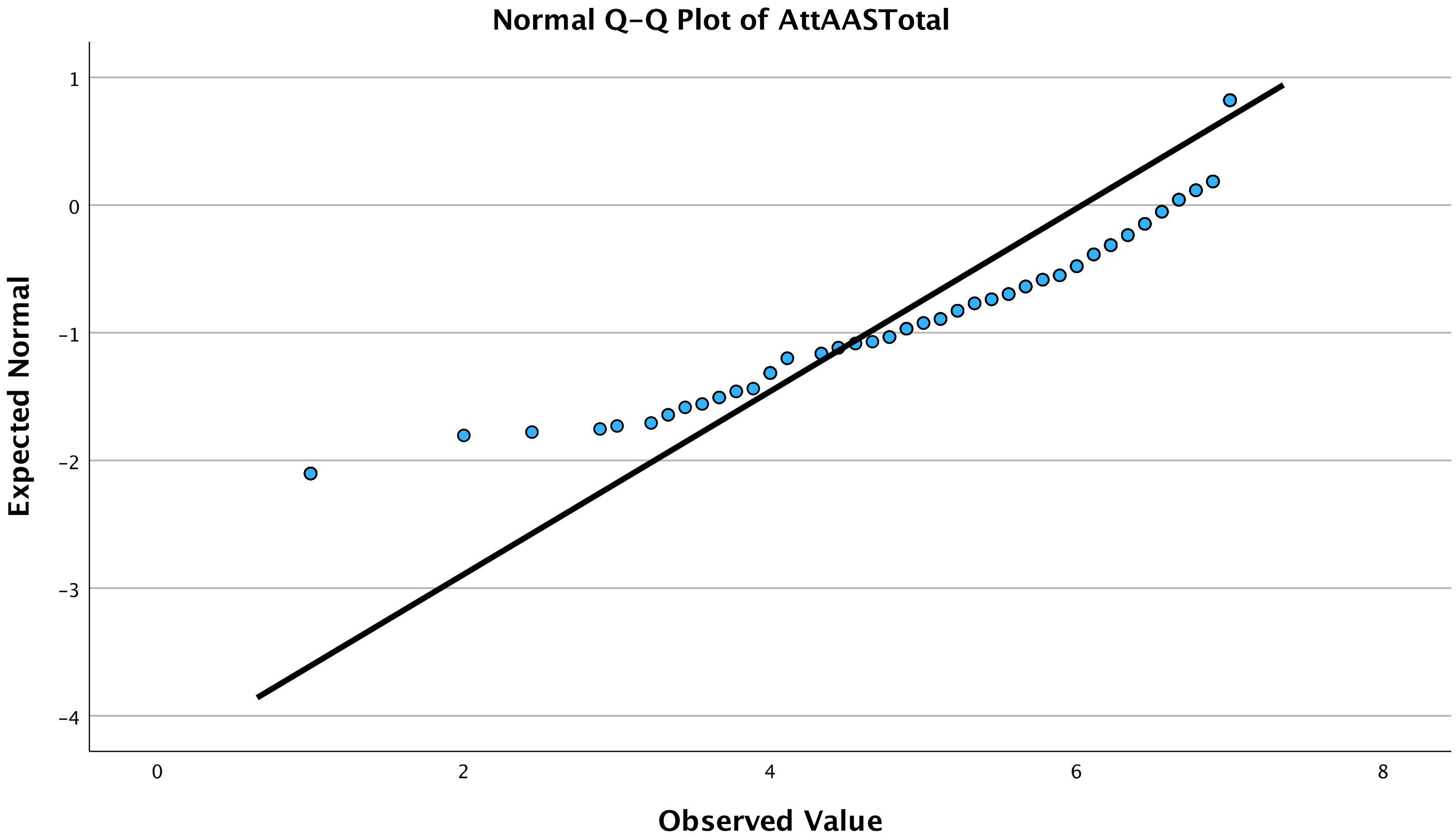
Normal Q-Q Plot of AttAASTotal
1
0
-1
-2
-3
-4
Expected Normal
0
2
4
6
8
Observed Value

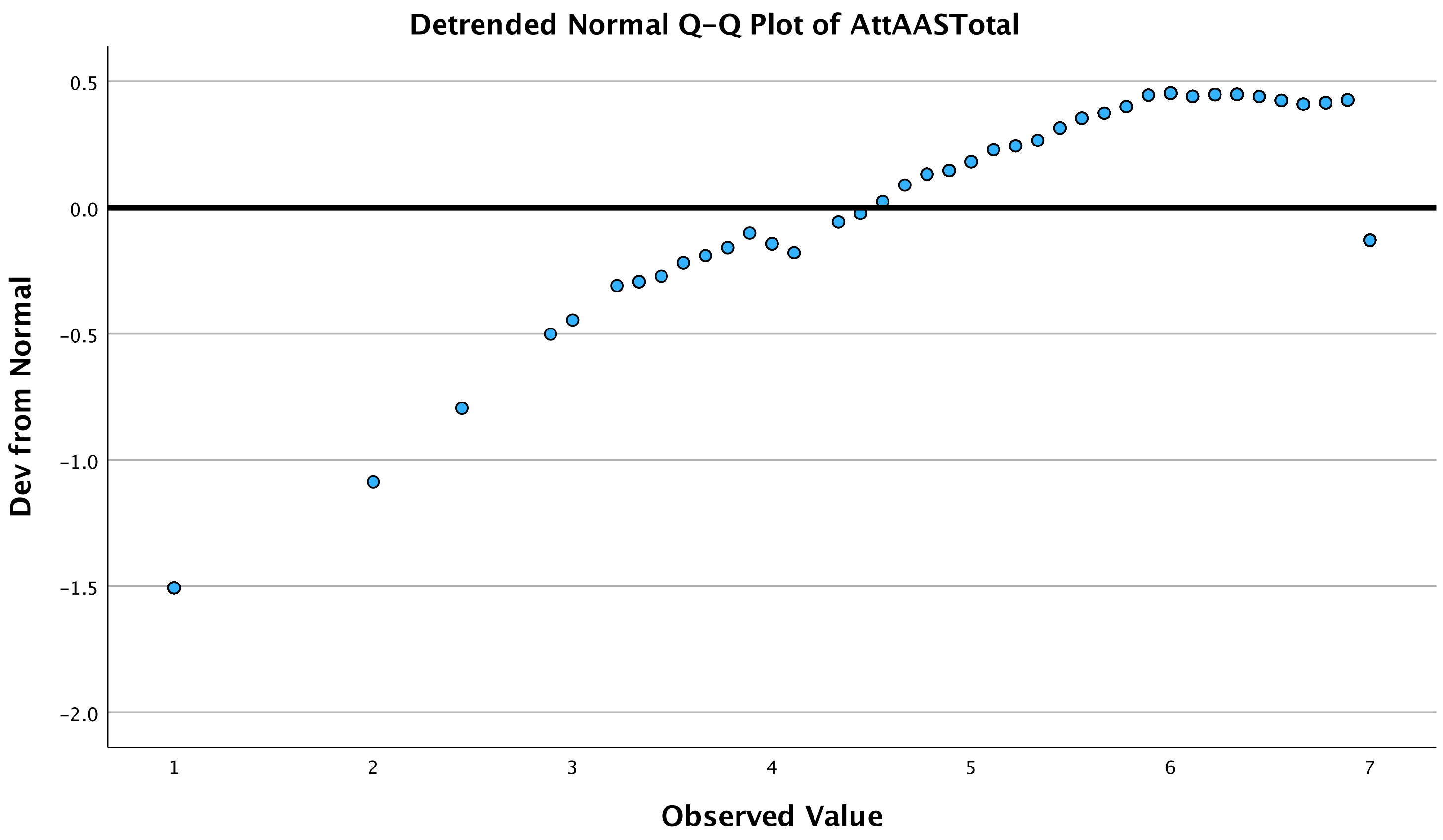

Detrended Normal Q-Q Plot of AttAASTotal
Dev from Normal
0.5
0.0
-0.5
-1.0
-1.5
-2.0
1
2
3
4
5
6
7
Observed Value

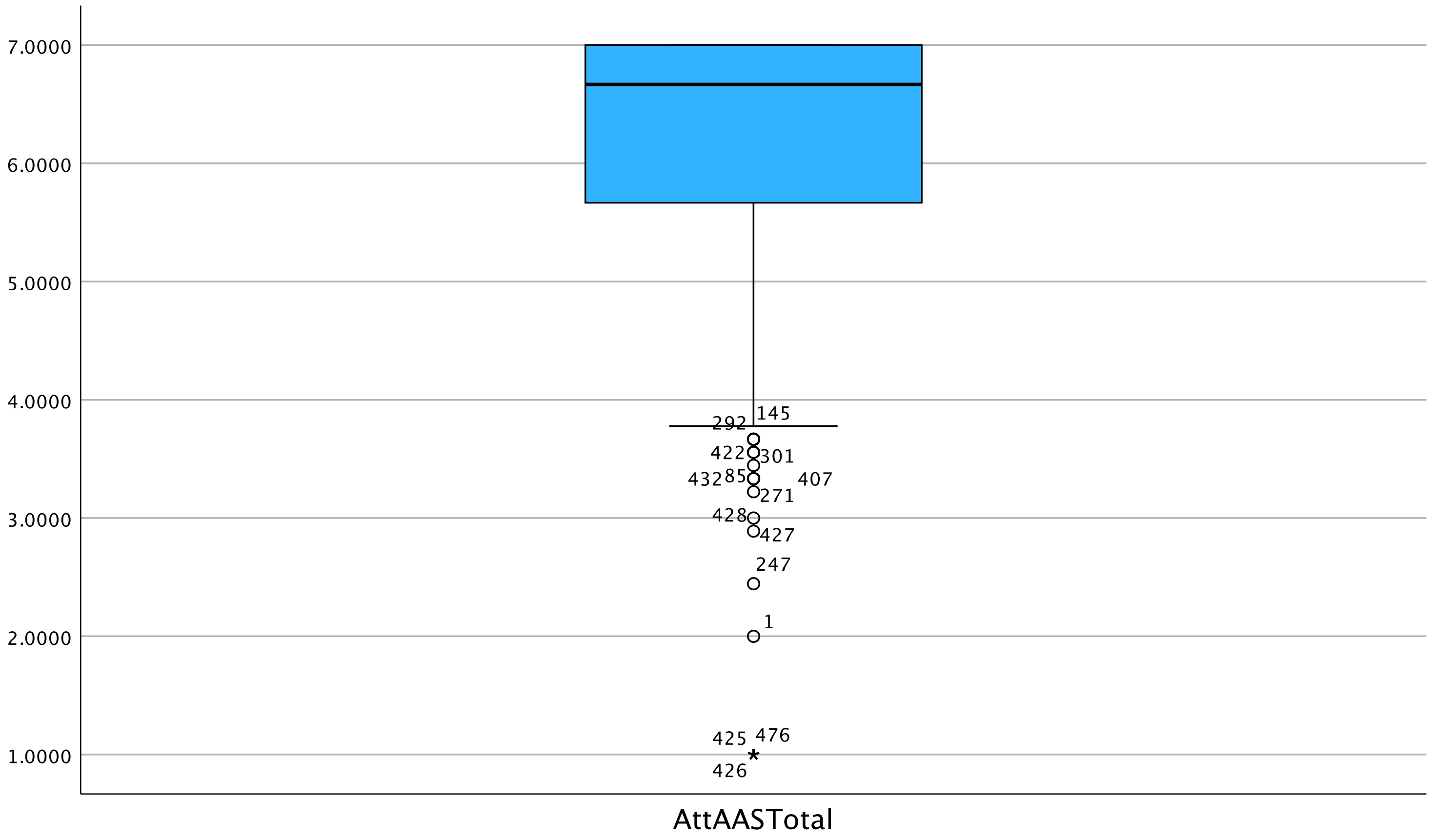
7.0000
6.0000
5.0000
4.0000
3.0000
2.0000
1.0000
145
292
422
301
432
85
407
271
428
427
247
1
425
476
426
AttAASTotal
