# Supplementary material for: Appearance and performance factors associated with muscle building supplement use and favourable attitudes towards anabolic steroids in adolescent boys
Source: Front Psychol. 2023 Sep 8;14:1241024. doi: 10.3389/fpsyg.2023.1241024 (PMC10516554; doi:10.3389/fpsyg.2023.1241024)
Supplement: Supplementary file 2 [file Data_Sheet_2.pdf]

MVA

Notes

Output Created		29-APR-2023 09:27:30
Comments		
Input	Data	/Users/joannadoley_1/Dropbox/Shared Folder (2)/Data/Data for Olivia/JoDataredo.sav
	Active Dataset	DataSet2
	Filter	<none>
	Weight	<none>
	Split File	<none>
	N of Rows in Working Data File	491
Syntax		MVA VARIABLES=AttAASTotal nosports_total weights MuscIdealTOTAL BodfatDisTOTAL MuscDisTOTAL /TPATTERN PERCENT=1 /EM(TOLERANCE=0.001 CONVERGENCE=0.0001 ITERATIONS=25

		OUTFILE='/Users/joannadoley_1/Dropbox/Shared Folder (2)/Data/Data for '+ 'Olivia/MissingValueUpdateAttitudes.sav').
Resources	Processor Time	00:00:00.13
	Elapsed Time	00:00:00.00
Files Saved	EM Imputed Data	/Users/joannadoley_1/Dropbox/Shared Folder (2)/Data/Data for Olivia/MissingValueUpdateAttitudes.sav

```
[DataSet2] /Users/joannadoley_1/Dropbox/Shared Folder (2)/Data/Data for Olivia/JoDataredo.sav
```

Univariate Statistics

	N	Mean	Std. Deviation	Missing		No. of Extremes[a]	
				Count	Percent	Low	High
AttAASTotal	477	6.036105	1.3956106	14	2.9	33	0
nosports_total	466	2.3863	1.54396	25	5.1	0	15
weights	491	.1446	.35206	0	.0	.	.
MuscIdealTOTAL	481	3.219854	.9120865	10	2.0	22	0
BodfatDisTOTAL	475	1.999579	1.0254565	16	3.3	0	10
MuscDisTOTAL	474	2.348101	.8339922	17	3.5	0	7

a. Number of cases outside the range (Q1 - 1.5*IQR, Q3 + 1.5*IQR).

Summary of Estimated Means

	AttAASTotal	nosports_total	weights	MuscIdealTOTAL	BodfatDisTOTAL	MuscDisTOTAL
All Values	6.036105	2.3863	.1446	3.219854	1.999579	2.348101
EM	6.037020	2.3843	.1446	3.220078	2.001599	2.349725

Summary of Estimated Standard Deviations

	AttAASTotal	nosports_total	weights	MuscIdealTOTAL	BodfatDisTOTAL	MuscDisTOTAL
All Values	1.3956106	1.54396	.35206	.9120865	1.0254565	.8339922
EM	1.3950949	1.54123	.35206	.9115198	1.0250342	.8324430

Tabulated Patterns

Number of Cases	Missing Patterns[a]						Complete if ...[b]
	weights	MuscIdealTOTAL	AttAASTotal	BodfatDisTOTAL	MuscDisTOTAL	nosports_total	
440							440
7				X			447
8					X		448
6		X					446
15						X	455

Patterns with less than 1% cases (5 or fewer) are not displayed.

a. Variables are sorted on missing patterns.

b. Number of complete cases if variables missing in that pattern (marked with X) are not used.

EM Estimated Statistics

EM Means[a]

AttAASTotal	nosports_total	weights	MuscIdealTOTAL	BodfatDisTOTAL	MuscDisTOTAL
6.037020	2.3843	.1446	3.220078	2.001599	2.349725

a. Little's MCAR test: Chi-Square = 67.688, DF = 39, Sig. = .003

EM Covariances[a]

	AttAASTotal	nosports_total	weights	MuscIdealTOTAL	BodfatDisTOTAL	MuscDisTOTAL
AttAASTotal	1.9462898					
nosports_total	-.0687347	2.37539				
weights	-.0303075	.13003	.12395			
MuscIdealTOTAL	-.2422610	.18012	.07791	.8308684		
BodfatDisTOTAL	-.3096284	-.11411	.04104	.1971211	1.0506951	
MuscDisTOTAL	-.2578387	.19834	.03724	.3557120	.3774683	.6929614

a. Little's MCAR test: Chi-Square = 67.688, DF = 39, Sig. = .003

EM Correlations[a]

	AttAASTotal	nosports_total	weights	MuscIdealTOTAL	BodfatDisTOTAL	MuscDisTOTAL
AttAASTotal	1					
nosports_total	-.032	1				
weights	-.062	.240	1			

MuscIdealTOTAL	-.191	.128	.243	1		
BodfatDisTOTAL	-.217	-.072	.114	.211	1	
MuscDisTOTAL	-.222	.155	.127	.469	.442	1

a. Little's MCAR test: Chi-Square = 67.688, DF = 39, Sig. = .003

Generalized Linear Models

Notes

Output Created		29-APR-2023 09:30:58
Comments		
Input	Data	/Users/joannadoley_1/Dropbox/Shared Folder (2)/Data/Data for Olivia/MissingValueUpdateAttitudes.sav
	Active Dataset	DataSet3
	Filter	<none>
	Weight	<none>
	Split File	<none>
	N of Rows in Working Data File	491
Missing Value Handling	Definition of Missing	User-defined missing values for factor, subject and within-subject variables are treated as

		missing.
	Cases Used	Statistics are based on cases with valid data for all variables in the model.
Weight Handling		not applicable
Syntax		GENLIN AttAASTotal BY nosports_total weights MuscIdealTOTAL BodfatDisTOTAL MuscDisTOTAL (ORDER=ASCENDING) /MODEL nosports_total weights MuscIdealTOTAL BodfatDisTOTAL MuscDisTOTAL INTERCEPT=YES DISTRIBUTION=GAMMA LINK=LOG /CRITERIA METHOD=FISHER(1) SCALE=MLE COVB=MODEL MAXITERATIONS=100 MAXSTEPHALVING=5 PCONVERGE=1E-006(ABSOLUTE) SINGULAR=1E-012 ANALYSISTYPE=1(WALD) CILEVEL=95 CITYPE=WALD

		LIKELIHOOD=FULL /MISSING CLASSMISSING=EXCLUDE /PRINT CPS DESCRIPTIVES MODELINFO FIT SUMMARY /SAVE XBPRED XBSTDERROR COOK LEVERAGE STDDEVIANCERESID.
Resources	Processor Time	00:00:00.55
	Elapsed Time	00:00:00.00
Variables Created or Modified	Predicted Value of the Linear Predictor	XBPredicted
	Estimated Standard Error of the Predicted Value of the Linear Predictor	XBStandardError
	Leverage Value	Leverage
	Standardized Deviance Residual	StdDevianceResidual
	Cook's Distance	CooksDistance

```
[DataSet3] /Users/joannadoley_1/Dropbox/Shared Folder (2)/Data/Data for
Olivia/MissingValueUpdateAttitudes.sav
```

Model Information

Dependent Variable	AttAASTotal
Probability Distribution	Gamma
Link Function	Log

Case Processing Summary

	N	Percent
Included	491	100.0%
Excluded	0	0.0%
Total	491	100.0%

Categorical Variable Information

			N	Percent
Factor	nosports_total	.00	12	2.4%
		1.00	121	24.6%
		1.89	1	0.2%
		2.00	164	33.4%
		2.05	1	0.2%
		2.06	1	0.2%
		2.16	1	0.2%
		2.19	1	0.2%
		2.21	1	0.2%
		2.23	4	0.8%
		2.25	1	0.2%
		2.27	1	0.2%
		2.28	1	0.2%
		2.31	1	0.2%

		2.33	1	0.2%
		2.37	1	0.2%
		2.39	1	0.2%
		2.41	1	0.2%
		2.47	1	0.2%
		2.48	1	0.2%
		2.50	1	0.2%
		2.64	1	0.2%
		2.67	1	0.2%
		2.85	1	0.2%
		2.99	1	0.2%
		3.00	81	16.5%
		4.00	56	11.4%
		5.00	17	3.5%
		6.00	9	1.8%
		7.00	1	0.2%
		8.00	3	0.6%
		9.00	1	0.2%
		17.00	1	0.2%
		Total	491	100.0%
	Weight training	.00	420	85.5%
		1.00	71	14.5%
		Total	491	100.0%
	Mesomorphic ideal internalisation Total	1.0000	13	2.6%
		1.2500	9	1.8%
		1.5000	8	1.6%
		1.7500	13	2.6%
		2.0000	21	4.3%
		2.2500	20	4.1%

		2.5000	30	6.1%
		2.7132	1	0.2%
		2.7500	26	5.3%
		2.9749	1	0.2%
		3.0000	61	12.4%
		3.1292	4	0.8%
		3.2065	1	0.2%
		3.2500	59	12.0%
		3.4149	1	0.2%
		3.5000	53	10.8%
		3.5531	1	0.2%
		3.7500	55	11.2%
		3.9292	1	0.2%
		4.0000	46	9.4%
		4.2500	25	5.1%
		4.5000	14	2.9%
		4.7500	14	2.9%
		5.0000	14	2.9%
		Total	491	100.0%
	Body Fat Dissatisfaction Total	1.0000	99	20.2%
		1.2000	49	10.0%
		1.4000	53	10.8%
		1.4649	1	0.2%
		1.5098	1	0.2%
		1.6000	45	9.2%
		1.8000	40	8.1%
		1.9428	1	0.2%
		1.9537	4	0.8%
		1.9802	1	0.2%

		2.0000	21	4.3%
		2.0191	1	0.2%
		2.0353	1	0.2%
		2.0898	1	0.2%
		2.1468	1	0.2%
		2.1993	1	0.2%
		2.2000	21	4.3%
		2.4000	24	4.9%
		2.4404	1	0.2%
		2.5860	1	0.2%
		2.6000	20	4.1%
		2.7563	1	0.2%
		2.8000	14	2.9%
		3.0000	16	3.3%
		3.2000	8	1.6%
		3.4000	13	2.6%
		3.6000	11	2.2%
		3.8000	6	1.2%
		4.0000	8	1.6%
		4.2000	4	0.8%
		4.4000	7	1.4%
		4.6000	6	1.2%
		4.8000	3	0.6%
		5.0000	7	1.4%
		Total	491	100.0%
	Muscularity Dissatisfaction Total	1.0000	26	5.3%
		1.1429	9	1.8%
		1.2857	9	1.8%
		1.4286	23	4.7%

	1.5714	35	7.1%
	1.6687	1	0.2%
	1.7143	29	5.9%
	1.8571	31	6.3%
	1.9482	1	0.2%
	2.0000	39	7.9%
	2.0947	1	0.2%
	2.1429	31	6.3%
	2.2400	1	0.2%
	2.2857	30	6.1%
	2.3063	4	0.8%
	2.3576	1	0.2%
	2.3710	1	0.2%
	2.4286	26	5.3%
	2.4437	1	0.2%
	2.5701	1	0.2%
	2.5714	23	4.7%
	2.5900	1	0.2%
	2.7143	23	4.7%
	2.7761	1	0.2%
	2.7826	1	0.2%
	2.8191	1	0.2%
	2.8279	1	0.2%
	2.8571	24	4.9%
	3.0000	41	8.4%
	3.1429	11	2.2%
	3.2857	11	2.2%
	3.4286	6	1.2%
	3.5714	7	1.4%

	3.7143	12	2.4%
	3.8571	7	1.4%
	4.0000	2	0.4%
	4.1429	4	0.8%
	4.2857	4	0.8%
	4.4286	4	0.8%
	4.5714	3	0.6%
	4.7143	1	0.2%
	5.0000	3	0.6%
	Total	491	100.0%

Continuous Variable Information

		N	Minimum	Maximum	Mean	Std. Deviation
Dependent Variable	AttAASTotal	491	1.0000	7.0000	6.037022	1.3759142

Goodness of Fit[a]

	Value	df	Value/df
Deviance	30.151	372	.081
Scaled Deviance	495.973	372	
Pearson Chi-Square	18.790	372	.051
Scaled Pearson Chi-Square	309.081	372	
Log Likelihood[b]	-873.094		
Akaike's Information Criterion (AIC)	1986.189		
Finite Sample Corrected AIC (AICC)	2064.675		
Bayesian Information	2489.762		

Criterion (BIC)			
Consistent AIC (CAIC)	2609.762		

Dependent Variable: AttAASTotal
Model: (Intercept), nosports_total, Weight training, Mesomorphic ideal internalisation Total, Body Fat Dissatisfaction Total, Muscularity Dissatisfaction Total
[a]

a. Information criteria are in smaller-is-better form.
b. The full log likelihood function is displayed and used in computing information criteria.

Omnibus Test[a]

Likelihood Ratio Chi-Square	df	Sig.
228.743	118	<.001

Dependent Variable: AttAASTotal
Model: (Intercept), nosports_total, Weight training, Mesomorphic ideal internalisation Total, Body Fat Dissatisfaction Total, Muscularity Dissatisfaction Total
[a]

a. Compares the fitted model against the intercept-only model.

Tests of Model Effects

Source	Type I		
	Wald Chi-Square	df	Sig.
(Intercept)	25589.543	1	<.001

nosports_total	144.798	32	<.001
Weight training	3.615	1	.057
Mesomorphic ideal internalisation Total	54.607	22	<.001
Body Fat Dissatisfaction Total	128.562	29	<.001
Muscularity Dissatisfaction Total	80.511	34	<.001

Dependent Variable: AttAASTotal
Model: (Intercept), nosports_total, Weight training, Mesomorphic ideal internalisation Total, Body Fat Dissatisfaction Total, Muscularity Dissatisfaction Total

Explore

Notes

Output Created		29-APR-2023 09:31:41
Comments		
Input	Data	/Users/joannadoley_1/Dropbox/Shared Folder (2)/Data/Data for Olivia/MissingValueUpdateAttitudes.sav
	Active Dataset	DataSet3
	Filter	<none>
	Weight	<none>

	Split File	<none>
	N of Rows in Working Data File	491
Missing Value Handling	Definition of Missing	User-defined missing values for dependent variables are treated as missing.
	Cases Used	Statistics are based on cases with no missing values for any dependent variable or factor used.
Syntax		EXAMINE VARIABLES=StdDevianceResidual /PLOT BOXPLOT HISTOGRAM NPPLOT /COMPARE GROUPS /STATISTICS DESCRIPTIVES /CINTERVAL 95 /MISSING LISTWISE /NOTOTAL.
Resources	Processor Time	00:00:02.17
	Elapsed Time	00:00:02.00

Case Processing Summary

Cases					
Valid		Missing		Total	
N	Percent	N	Percent	N	Percent

Standardized Deviance Residual	466	94.9%	25	5.1%	491	100.0%

Descriptives

			Statistic	Std. Error
Standardized Deviance Residual	Mean		-.08829	.052217
	95% Confidence Interval for Mean	Lower Bound	-.19090	
		Upper Bound	.01432	
	5% Trimmed Mean		.02889	
	Median		.08746	
	Variance		1.271	
	Std. Deviation		1.127218	
	Minimum		-6.011	
	Maximum		2.424	
	Range		8.435	
	Interquartile Range		.834	
	Skewness		-2.782	.113
	Kurtosis		11.237	.226

Tests of Normality

	Kolmogorov-Smirnov[a]			Shapiro-Wilk		
	Statistic	df	Sig.	Statistic	df	Sig.
Standardized Deviance Residual	.187	466	<.001	.752	466	<.001

a. Lilliefors Significance Correction

Standardized Deviance Residual

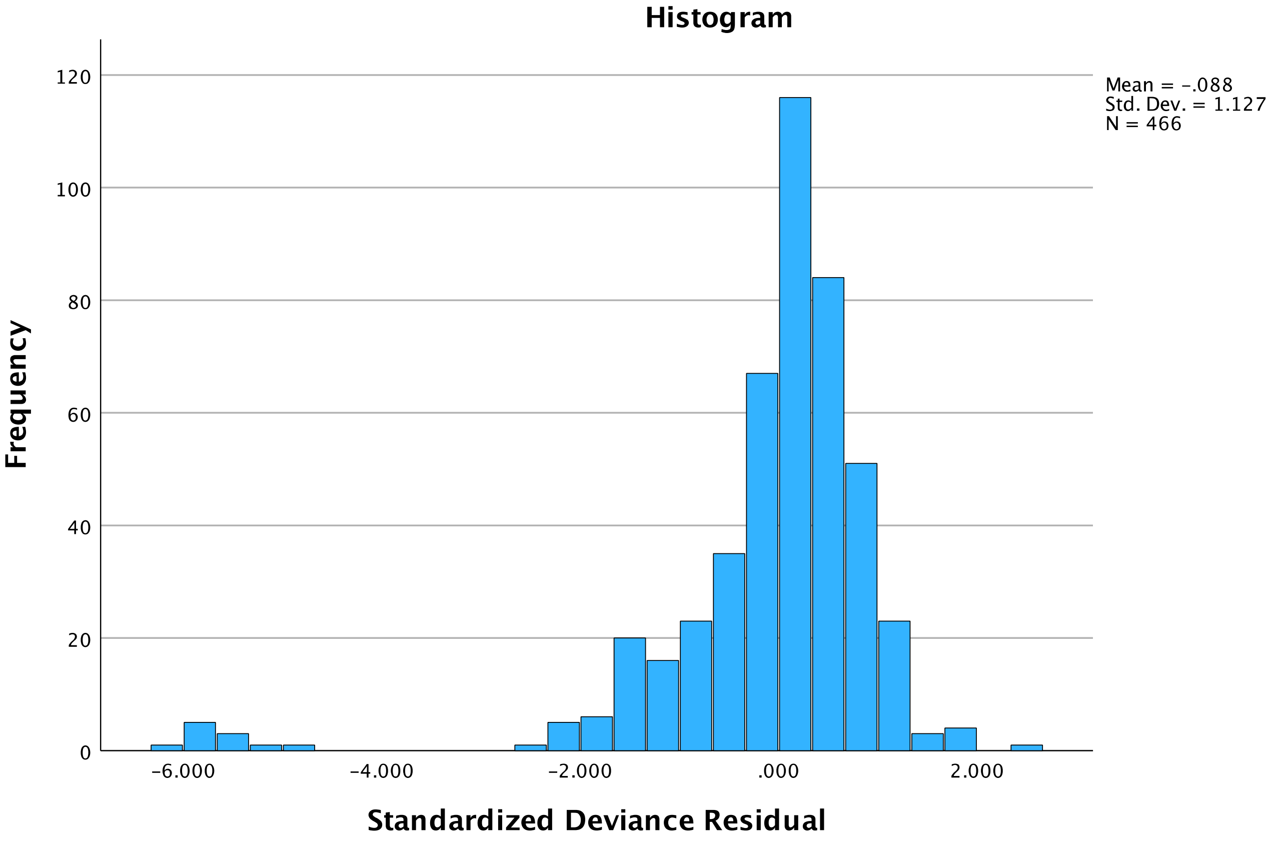

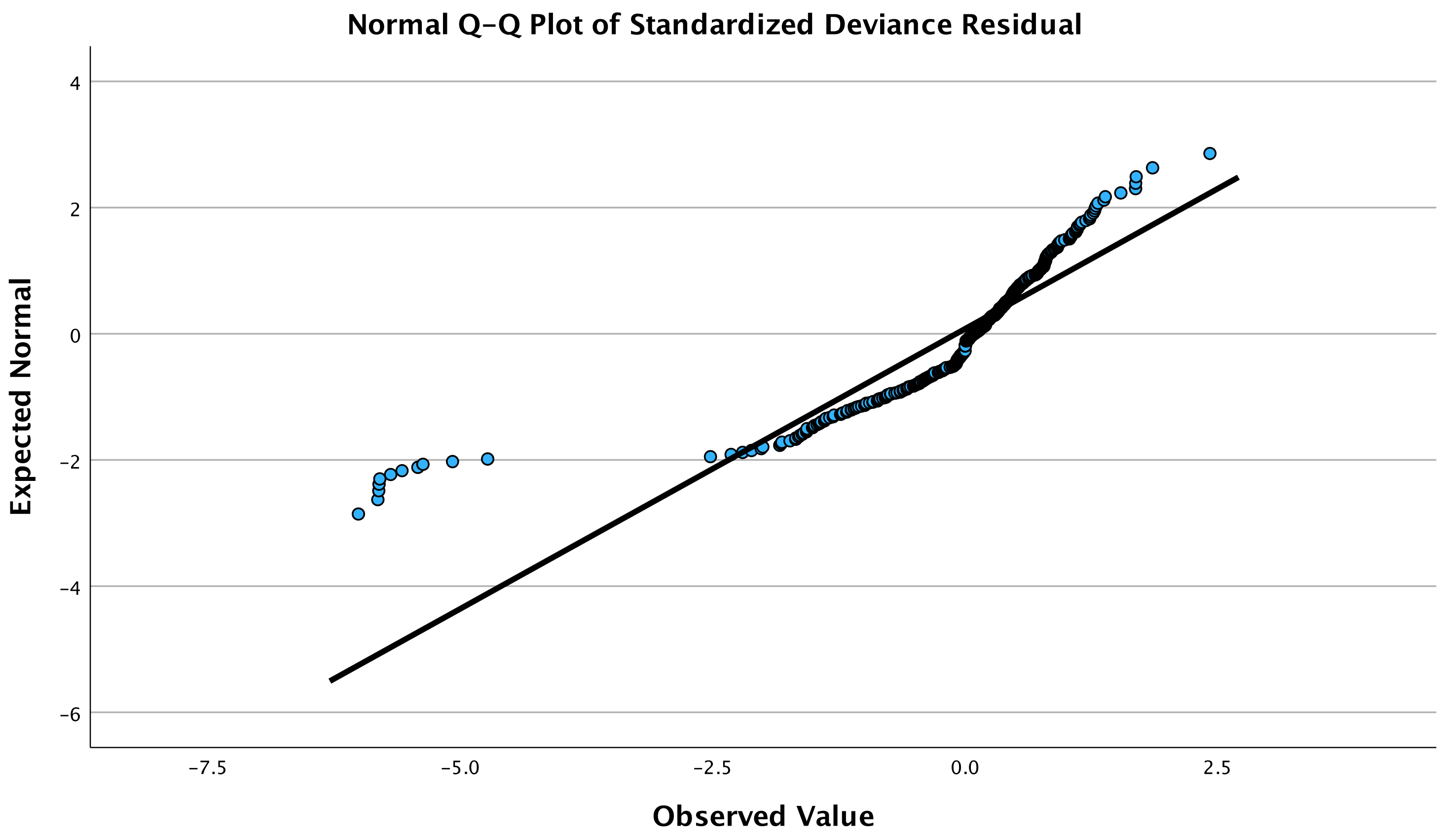

Normal Q-Q Plot of Standardized Deviance Residual
Expected Normal
4
2
0
-2
-4
-6
-7.5
-5.0
-2.5
0.0
2.5
Observed Value

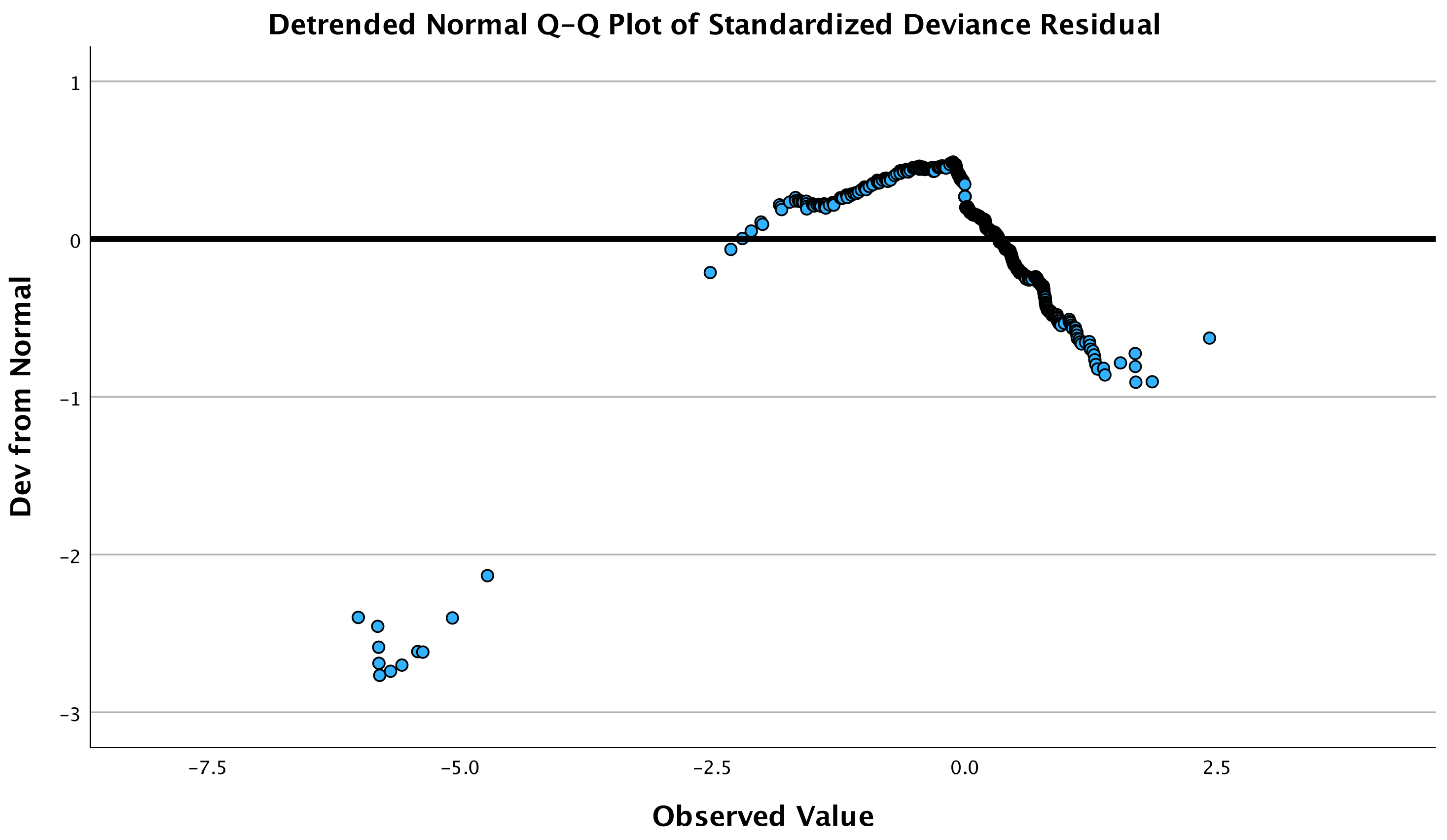

Detrended Normal Q-Q Plot of Standardized Deviance Residual
Dev from Normal
1
0
-1
-2
-3
-7.5
-5.0
-2.5
0.0
2.5
Observed Value

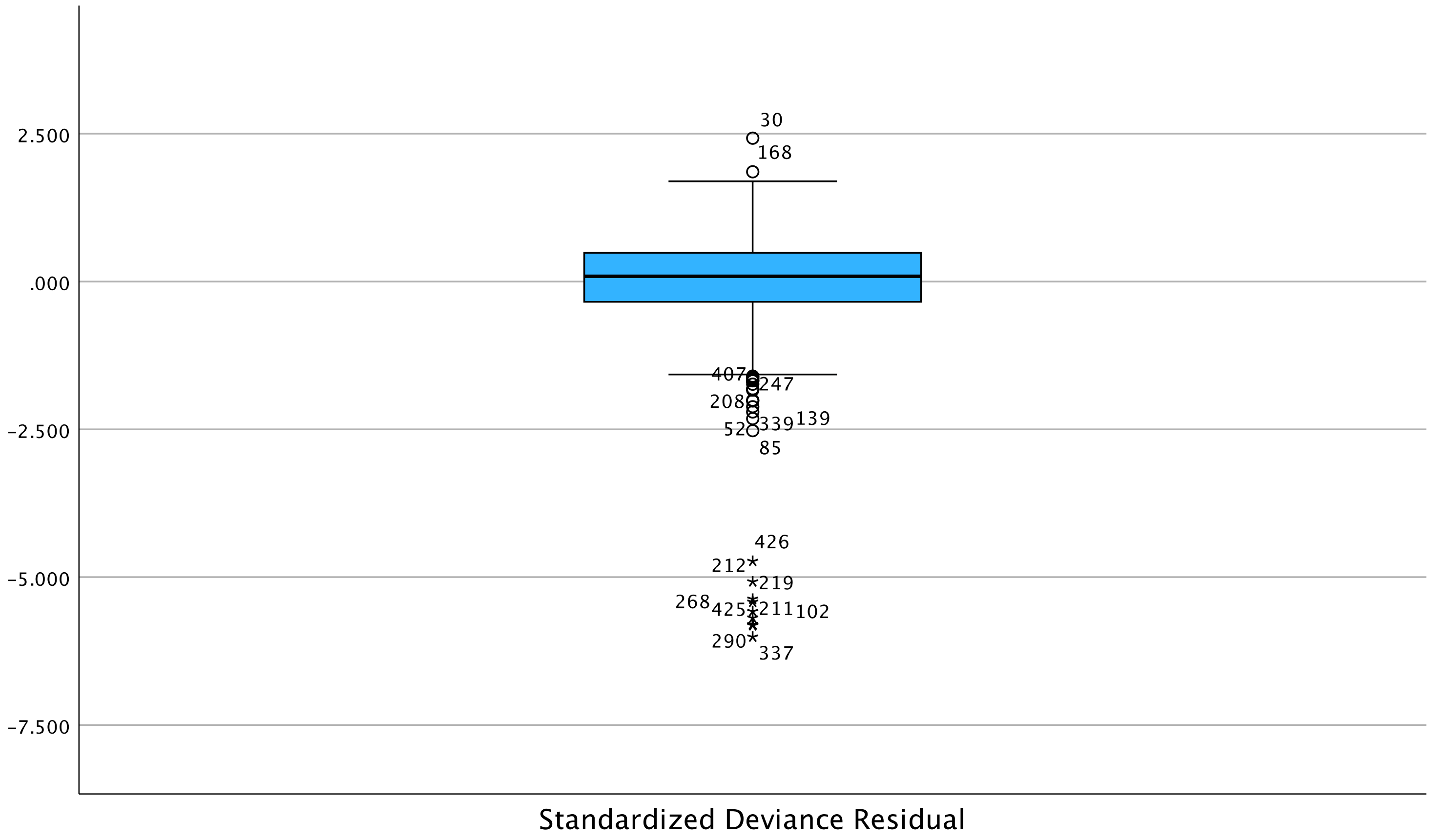

GGraph

Notes	
Output Created	29-APR-2023 12:16:49

Comments		
Input	Data	/Users/joannadoley_1/Dropbox/Shared Folder (2)/Data/Data for Olivia/MissingValueUpdateAttitudes.sav
	Active Dataset	DataSet3
	Filter	<none>
	Weight	<none>
	Split File	<none>
	N of Rows in Working Data File	491
Syntax		GGRAPH /GRAPHDATASET NAME="graphdataset" VARIABLES=StdDevianceResidual XBPredicted MISSING=LISTWISE REPORTMISSING=NO /GRAPHSPEC SOURCE=INLINE /FITLINE TOTAL=NO SUBGROUP=NO. BEGIN GPL GUIDE: axis(dim(1), label("Standardized Deviance Residual")) GUIDE: axis(dim(2), label("Predicted Value of Linear Predictor")) GUIDE:

		text.title(label("Scatter Plot of Predicted Value of Linear Predictor by Standardized ", "Deviance Residual")) ELEMENT: point(position(StdDevianceResidual*XBPredicted)) END GPL.
Resources	Processor Time	00:00:00.76
	Elapsed Time	00:00:01.00

Scatter Plot of Predicted Value of Linear Predictor by Standardized Deviance Residual

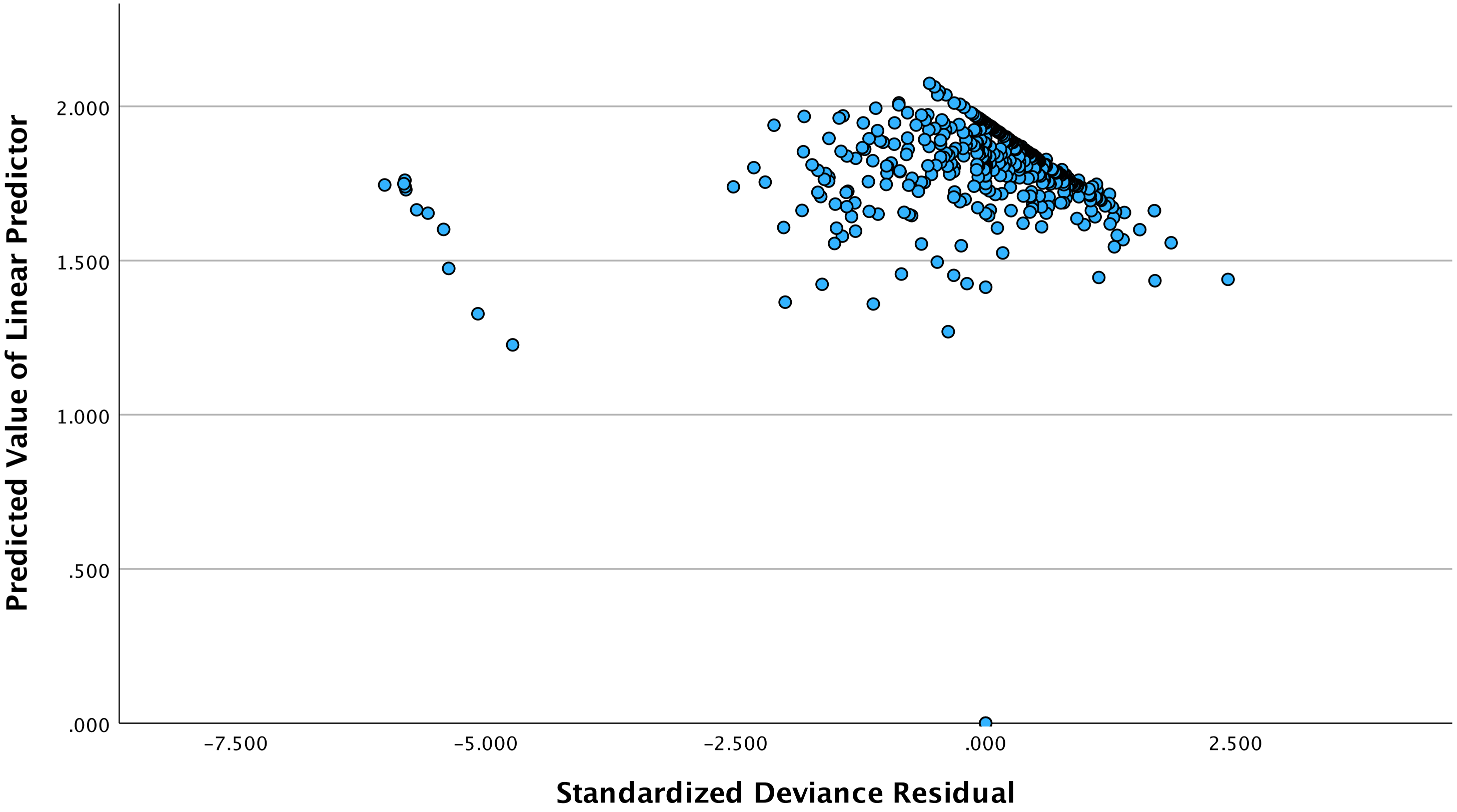

Regression

Notes

Output Created	29-APR-2023 12:18:44

Comments		
Input	Data	/Users/joannadoley_1/Dropbox/Shared Folder (2)/Data/Data for Olivia/MissingValueUpdateAttitudes.sav
	Active Dataset	DataSet3
	Filter	<none>
	Weight	<none>
	Split File	<none>
	N of Rows in Working Data File	491
Missing Value Handling	Definition of Missing	User-defined missing values are treated as missing.
	Cases Used	Statistics are based on cases with no missing values for any variable used.
Syntax		REGRESSION /MISSING LISTWISE /STATISTICS COEFF OUTS R ANOVA COLLIN TOL /CRITERIA=PIN(.05) POUT(.10) /NOORIGIN /DEPENDENT AttAASTotal /METHOD=ENTER nosports_total weights

		MuscIdealTOTAL BodfatDisTOTAL MuscDisTOTAL.
Resources	Processor Time	00:00:00.03
	Elapsed Time	00:00:00.00
	Memory Required	5008 bytes
	Additional Memory Required for Residual Plots	0 bytes

Variables Entered/Removed[a]

Model	Variables Entered	Variables Removed	Method
1	Muscularity Dissatisfaction Total, Weight training, nosports_total, Body Fat Dissatisfaction Total, Mesomorphic ideal internalisation Total[b]	.	Enter

a. Dependent Variable: AttAASTotal
b. All requested variables entered.

Model Summary

Model	R	R Square	Adjusted R	Std. Error of

			Square	the Estimate
1	.279[a]	.078	.069	1.3279052

a. Predictors: (Constant), Muscularity Dissatisfaction Total, Weight training, nosports_total, Body Fat Dissatisfaction Total, Mesomorphic ideal internalisation Total

ANOVA[a]

Model		Sum of Squares	df	Mean Square	F	Sig.
1	Regression	72.422	5	14.484	8.214	<.001[b]
	Residual	855.216	485	1.763		
	Total	927.639	490			

a. Dependent Variable: AttAASTotal
b. Predictors: (Constant), Muscularity Dissatisfaction Total, Weight training, nosports_total, Body Fat Dissatisfaction Total, Mesomorphic ideal internalisation Total

Coefficients[a]

Model		Unstandardized Coefficients		Standardized Coefficients	t	Sig.	Collinearity Statistics
		B	Std. Error	Beta			Tolerance
1	(Constant)	7.421	.255		29.135	<.001	
	nosports_total	-.012	.042	-.013	-.274	.784	.892
	Weight training	-.007	.181	-.002	-.041	.967	.884
	Mesomorphic ideal internalisation Total	-.167	.077	-.110	-2.165	.031	.739
	Body Fat Dissatisfaction Total	-.205	.068	-.150	-3.025	.003	.769
	Muscularity	-.173	.092	-.103	-1.881	.061	.629

	Dissatisfaction Total						

Coefficients[a]

Model		Collinearity Statistics VIF
1	(Constant)	
	nosports_total	1.121
	Weight training	1.132
	Mesomorphic ideal internalisation Total	1.352
	Body Fat Dissatisfaction Total	1.300
	Muscularity Dissatisfaction Total	1.591

a. Dependent Variable: AttAASTotal

Collinearity Diagnostics[a]

Model	Dimension	Eigenvalue	Condition Index	Variance Proportions			
				(Constant)	nosports_total	Weight training	Mesomorphic ideal internalisation Total
1	1	4.743	1.000	.00	.01	.01	.00
	2	.789	2.452	.00	.00	.89	.00
	3	.269	4.197	.00	.66	.04	.00
	4	.112	6.519	.05	.29	.01	.12

	5	.055	9.298	.28	.00	.00	.02
	6	.033	12.036	.67	.04	.06	.86

Collinearity Diagnostics[a]

Model	Dimension	Variance Proportions: Body Fat Dissatisfaction Total	Variance Proportions: Muscularity Dissatisfaction Total
1	1	.01	.00
	2	.00	.00
	3	.15	.00
	4	.69	.01
	5	.08	.84
	6	.07	.13

a. Dependent Variable: AttAASTotal
